# Self-reported Work Ability and Work Performance in Workers with Chronic Nonspecific Musculoskeletal Pain

**DOI:** 10.1007/s10926-012-9373-1

**Published:** 2012-06-03

**Authors:** Haitze J. de Vries, Michiel F. Reneman, Johan W. Groothoff, Jan H. B. Geertzen, Sandra Brouwer

**Affiliations:** 1 Department of Rehabilitation Medicine, Center for Rehabilitation, University Medical Center Groningen, University of Groningen, P.O. Box 30.002, 9750 RA Haren, The Netherlands; 2 Department of Health Sciences, Community and Occupational Medicine, University Medical Center Groningen, University of Groningen, Groningen, The Netherlands

**Keywords:** Work ability, Work performance, Chronic pain, Musculoskeletal disorders, Staying at work

## Abstract

*Purpose* To assess self-reported work ability and work performance of workers who stay at work despite chronic nonspecific musculoskeletal pain (CMP), and to explore which variables were associated with these outcomes. *Methods* In a cross-sectional study we assessed work ability (Work Ability Index, single item scale 0–10) and work performance (Health and Work Performance Questionnaire, scale 0–10) among 119 workers who continued work while having CMP. Scores of work ability and work performance were categorized into excellent (10), good (9), moderate (8) and poor (0–7). Hierarchical multiple regression and logistic regression analysis was used to analyze the relation of socio-demographic, pain-related, personal- and work-related variables with work ability and work performance. *Results* Mean work ability and work performance were 7.1 and 7.7 (poor to moderate). Hierarchical multiple regression analysis revealed that higher work ability scores were associated with lower age, better general health perception, and higher pain self-efficacy beliefs (R^2^ = 42 %). Higher work performance was associated with lower age, higher pain self-efficacy beliefs, lower physical work demand category and part-time work (R^2^ = 37 %). Logistic regression analysis revealed that work ability ≥8 was significantly explained by age (OR = 0.90), general health perception (OR = 1.04) and pain self-efficacy (OR = 1.15). Work performance ≥8 was explained by pain self-efficacy (OR = 1.11). *Conclusions* Many workers with CMP who stay at work report poor to moderate work ability and work performance. Our findings suggest that a subgroup of workers with CMP can stay at work with high work ability and performance, especially when they have high beliefs of pain self-efficacy. Our results further show that not the pain itself, but personal and work-related factors relate to work ability and work performance.

## Introduction

Chronic nonspecific musculoskeletal pain (CMP) accounts for large costs to society [1, 2]. Many workers with CMP report decreased work ability or work performance, which impairs their work productivity [3, 4] and may lead to long-term sickness absence and work disability. However, although many workers with CMP discontinue work, most workers are able to cope with CMP and still attend work while having pain [5, 6]. It is under debate whether remaining at work with chronic pain is wise: it may adversely affect health [7] and the question is whether these workers remain productive. Therefore, it is of importance to focus research not only on highly disabled or sick-listed groups, but also on its successful counterpart [8] and to learn which factors are associated with work ability and work performance in workers who stay at work with CMP.

To investigate the workers’ ability to participate in work, the concept of work ability has been introduced. It is defined as the degree to which a worker, given his health, is physically and mentally able to cope with the demands at work [9]. High associations between work ability and productivity loss due to absenteeism have been observed [10, 11]. Likewise, two recent studies on work productivity showed that having pain is associated with higher levels of reduced work performance [4, 12]. Reduced work performance accounts largely for indirect costs due to productivity loss [13, 14]. When work productivity is affected by reduced performance due to a health problem, it is often referred to as presenteeism. In recent years, it has been demonstrated that societal costs related to CMP are not only related to absenteeism, but to presenteeism as well [14, 15]. The costs related to presenteeism might even exceed the costs of absenteeism [16–18].

In earlier research, different variables were observed to be associated with self-reported work ability or work performance in people with chronic pain conditions: age [10], gender [19], pain intensity [4, 20], general health perception [10, 21], fear avoidance [22], pain self-efficacy [23, 24], work demands [25, 26], number of working hours [26, 27], control over work tasks [28], and work satisfaction [27]. So far, knowledge of work ability and work performance focusing on people who stay at work despite CMP remains scarce. In the present study we connected to the existing knowledge on work ability and work performance, and focused on workers who stay at work despite CMP. Although this group of workers may be successful in terms of low absenteeism, their levels of work ability and work performance remain unclear. Moreover, knowledge about which variables are associated with high work ability and work performance despite CMP might help us to tailor vocational rehabilitation programs that prevent unneeded work disability and maintain work performance.

The aim of this study was twofold: to assess levels of self-reported work ability and work performance in people who stay at work with CMP, and to explore which socio-demographic, pain related, personal and work-related variables were associated with work ability and work performance.

## Methods

### Subjects

Participants in the “Working with Pain” study were recruited from May 2009 to December 2010 by announcements in newspapers, complemented with a call on the websites of national patient associations of low back pain, whiplash and fibromyalgia. It was made clear that they participated in scientific research and that no treatment or advice would be provided. A compensation of €50 and traveling compensation was offered for participation. Inclusion criteria of the “Working with Pain” study were: CMP (pain in back, neck, shoulder, extremities or disorders such as widespread pain, fibromyalgia and whiplash) without known underlying specific medical cause (e.g. infection, neoplasm, metastasis, osteoporosis, rheumatoid arthritis, fracture, neurological disorders, and serious spinal pathology); duration longer than 6 months; age 20–60 years; having been employed 20 h a week or more during 12 months prior to participation in the study. Participants’ absence from work ascribed to CMP could not be more than 5 % of potential total working hours in the 12 months prior to participation. The 5 % was chosen because it is around the average rate of sickness absence in The Netherlands and Europe [29, 30]. Exclusion criteria in this study were the following: hypertension or cardiovascular diseases, co-morbidities with severe negative consequences for physical and/or mental functioning (e.g. severe psychiatric disease or addiction to drugs), pregnancy, and insufficient knowledge of the Dutch language.

Sample size was determined by the amount of independent variables we intended to include into a logistic model. A minimum of 10 subjects per independent variable has been recommended [31]. Because we estimated to use 10 predicting variables in the model, a total sample size of at least 100 was needed.

### Procedure

To diagnose the type of pain and the existence of co-morbidities, all participants were medically examined by a physiatrist. All participants completed questionnaires assessing socio-demographic characteristics, work characteristics (work ability, work performance, relation with colleagues, relation with supervisor, work satisfaction, control over work tasks), pain related characteristics (pain region, pain intensity, pain disability), and personal characteristics (general health perception, fear avoidance beliefs, pain self-efficacy). In earlier research, these variables were observed to be associated with work ability or work performance [4, 10, 19, 21, 22, 26, 27]. The study was approved by the Medical Ethical Committee of the University Medical Center of Groningen. Anonymity, confidentiality, and the right to withdraw from the study at all times were guaranteed. All participants gave informed consent.

### Main Measures

Work ability was assessed with a single item of the Work Ability Index (WAI). Current work ability compared to lifetime best was scored on a 0–10 response scale, where 0 represents “completely unable to work” and 10 “work ability at its best”. A very strong association between this single WAI-item and the complete WAI was found [32]. The scores are categorized into excellent (score 10), good (score 9), moderate (score 8) and poor (score 0–7) [33, 34]. It was concluded that the single-item question could be used as a simple indicator for assessing self-reported work ability [32].

Work performance was assessed with the World Health Organization’s Health and Work Performance Questionnaire (HPQ). The HPQ is a reliable and valid self-rated work performance measure, scored as percentage of performance on a 0–10 response scale, where 0 represents a total lack of performance and 10 no lack of performance during time of the job in the past 4 weeks [35, 36]. The scores were categorized into excellent (score 10), good (score 9), moderate (score 8) and poor (score 0–7), adapted from Kessler et al. [35].

### Independent Variables and Covariates

*Socio*-*demographic characteristics* were recorded by a questionnaire constructed by Rehabilitation Development Centers in the Netherlands [37].

*Pain*-*related characteristics:* Diagnosis region, duration of pain and use of pain medication were recorded by a questionnaire constructed by Rehabilitation Development Centers in the Netherlands [37]. Pain intensity was measured using the 11-point numeric rating scale (NRS), ranging from 0 (no pain) to 10 (worst possible pain), requiring participants to rate their current pain intensity and average pain intensity [38]. Validity and utility of the 11-point NRS is sufficient and it is responsive to changes in individuals [39, 40].

The Pain Disability Index (PDI) was used to measure the degree to which chronic pain interferes with daily activities (self-perceived disability) [41, 42]. The PDI is a 7-item inventory, with each item being scored from 0 (no interference) to 10 (total interference). Higher scores reflect higher interference of pain with daily activities. The reliability and validity of the PDI is sufficient [41, 42].

*Personal characteristics:* The Dutch version of the RAND 36-item Health survey (RAND-36) was used to measure general health perception [43]. Scores range from 0 to 100, and higher scores reflect better perceived general health perception. The Dutch version of the RAND 36-items is a reliable, valid and sensitive instrument [43]. Fear avoidance beliefs about physical activity and (re)injury was measured with the Dutch version of the Tampa Scale of Kinesiophobia (TSK; 17 items) [44, 45]. Higher scores reflect higher perceived fear of physical activity. Reliability and validity of the Dutch version are good [44, 46]. Pain self-efficacy was measured by the Dutch version of the Pain Self Efficacy Questionnaire (PSEQ; 10 items) [47]. Each item is rated by selecting a number on a 7-point scale, scores ranging from 0 (“not at all confident”) to 6 (“completely confident”). Higher scores reflect stronger self-efficacy beliefs. Self-efficacy beliefs for people experiencing chronic pain incorporate not just the expectation that a person could perform a particular behavior or task, but also their confidence in being able to do it despite their pain [48]. The PSEQ has strong psychometric properties and high reliability and validity [48].

*Work characteristics:* Sick leave during the previous 12 months, full-time or part-time employment, and own prognosis to fulfill work 2 years from now were assessed by the WAI. The reliability and validity of the WAI are acceptable [9, 49]. Control over work tasks, social support at work, and work satisfaction were assessed by the Dutch questionnaire on the Perception and Evaluation of Work (Dutch abbreviation: VBBA) [50]. Subscale scores range between 0 and 100; higher scores indicate more unfavorable situations. The reliability and unidimensionality of all scales of the VBBA were considered satisfactory [50].

The physical work demand category was assessed according to the Dictionary of Occupational Titles (DOT). Within the DOT, occupations are classified into five categories of physical workload, based on intensity and duration of lifting or carrying needed for the job: sedentary, light, medium, heavy/very heavy [51]. The 5th DOT-category hardly exists in the Netherlands, because the Dutch laws on worker safety advise a maximum lifting weight of 23 kg. Therefore, in the present study the DOT-categories “heavy” and “very heavy” were combined into one. Validity of the DOT has not been scientifically tested nor has it been based on quantitative work-related task analyses, but rather on consensus meetings of experts [52, 53].

### Statistical Analysis

All statistical analyses were performed using SPSS for Windows, version 18.0.3 [54]. To answer what levels of work ability and performance were observed in workers with CMP, average scores with standard deviations, medians with interquartile range, and percentiles were provided. To answer which variables were associated with work ability and work performance, a hierarchical multiple regression analysis was used, with work ability and work performance as dependent variable. Candidate predictor variables were entered stepwise into the regression model: age (years), gender (female = 0, male = 1), pain intensity [20], general health perception [10, 21], fear avoidance [22], pain self-efficacy [23, 55], DOT-category (1–4) [26], employment (part-time = 0, full-time = 1), control over work tasks [28], and work satisfaction [27]. DOT-categories were entered as dummy variables in the regression equation. Beta values with 95 % confidence interval, standardized β and *p* values for all variables were calculated. For each step in the model, explained variance (R^2^ and R^2^-change) were calculated.

Logistic regression was applied to assess which of the independent variables were associated with high work ability and high work performance in workers with CMP. Therefore, work ability and work performance were transformed into dichotomous variables: scores on the single WAI item “current work ability compared to lifetime best” <8 were considered as low work ability, and scores ≥8 were considered as high work ability [32, 34]; scores on the HPQ-work-performance scale <8 were considered as low work performance, and scores ≥8 were considered as high work performance [19, 27, 35]. In all analyses a *p* value <0.05 was considered significant. Listwise deletion was used to discard the cases with missing values from the regression analysis.

## Results

A total of 119 subjects was included in the “Working with Pain” study. Detailed descriptive data of the participants are presented in Table 1. All potential participants were examined for eligibility: seven were not included in the study because of heart disease, high blood pressure, neurological disorder, radiculopathy and co-morbidity. Various potential participants registered for the study, but were not confirmed eligible because of age >60 years, specific medical cause such as rheumatoid arthritis, unpaid job, employment less than 20 h, or more than 5 % sick leave.

**Table 1:** Description of the study population, workers who stay at work with CMP (n = 119)

Variables	Range		Median [IQR]
*Socio*-*demographic characteristics*			
Age (years), mean (SD)		48.3 (7.8)	51 [44–55]
Gender male (%)		40	
Married/co-habitation (%)		90	
Educational level (%)			
Low		11	
Medium		56	
High		33	
*Pain*-*related characteristics*			
Pain region (%)			
Low back		53	
Neck/shoulders		13	
Fibromyalgia		23	
Other^a^		11	
Duration of pain (%)			
1–2 years		8	
2–5 years		11	
>5 years		81	
Pain medication (yes) ( %)		40	
NRS current pain^b^, mean (SD)	0–10	4.6 (2.1)	5 [3–6]
NRS worst pain, mean (SD)	0–10	6.9 (1.8)	7 [6–8]
PDI^c^, mean (SD)	0–70	19.9 (11.1)	19 [12–28]
*Personal characteristics*			
RAND 36 General health perception^d^, mean (SD)	0–100	62.9 (17.7)	65 [50–75]
Fear avoidance beliefs TSK^e^, mean (SD)	17–68	33.0 (7.2)	32 [28–39]
Pain self-efficacy beliefs PSEQ^f^, mean (SD)	0–60	46.9 (8.5)	49 [42–53]
*Work characteristics*			
Expected to work last week (hours), mean (SD)		31.5 (7.8)	
Actually worked last week (hours), mean (SD)		32.5 (10.4)	
Employment full-time (%)		50.4	
Physical demand category work^g^ (%)			
DOT 1 sedentary		35	
DOT 2 light		35	
DOT 3 moderate		24	
DOT 4 (very) heavy		6	
Relation with colleagues^h^, mean (SD)	0–100	7.1 (11.9)	0 [0–11]
Relation with supervisor^h^, mean (SD)	0–100	10.0 (20.0)	0 [0–11]
Work satisfaction^h^, mean (SD)	0–100	11.1 (18.8)	0 [0–11]
Control over work tasks^h^, mean (SD)	0–100	25.4 (26.5)	18 [0–36]
*Main outcome measures*			
Work ability single item (WAI)^i^, mean (SD)	0–10	7.1 (1.6)	7 [6–8]
Poor (0–7), %		57	
Moderate (8), %		25	
Good (9), %		10	
Excellent (10), %		8	
Work ability related to the demands of job, mean (SD)	2–10	7.6 (1.0)	8 [7–8]
Estimated work impairment due to CMP, mean (SD)	1–6	3.9 (1.2)	4 [3–5]
Sick leave during the past 12 months, mean (SD)^j^	1–5	4.6 (0.5)	5 [4–5]
Personal prognosis of work ability about 2 years, mean (sd)	1, 4 or 7	6.2 (1.4)	7 [4–7]
Work performance (HPQ)^k^, mean (SD)	0–10	7.7 (1.1)	8 [7–8]
Poor (0–7), %		30	
Moderate (8), %		50	
Good (9), %		17	
Excellent (10), %		3	
Relative presenteeism, mean (SD)	0.25-2	1.1 (0.3)	1 [1-1]

^a^Pain of extremity, cervical-brachial syndrome, generalized pain, ^b^ Numeric Rating Scale (0 = no pain, 10 = worst possible pain), ^c ^Pain Disability Index, ^d^ RAND 36-item Health Survey, ^e^ Tampa Scale for Kinesiophobia, ^f^ Pain Self Efficacy Questionnaire, ^g^ dictionary of occupational titles, ^h^ Subscale of Questionnaire on the Perception and Evaluation of Work (in Dutch: VBBA), ^i^ Work Ability Index, ^j^ Subscale of the WAI: 1 = ≥ 100 days sick leave; 4 = 1–10 day sick leave; 5 = no sick leave during the past 12 months, ^k^ Health and Work Performance Questionnaire

### Levels of Work Ability and Work Performance

The mean work ability level was 7.1 (SD 1.6), 43 % reported a work ability ≥8 (Table 1). The mean work performance level was 7.7 (SD 1.1). Work performance was rated ≥8 by 70 % of the subjects. Only 3 % of these workers reported the maximum score, which represents a top work performance. Eighty-one percent of the workers rated their work performance equal or better compared to their co-workers.

### Associations with Work Ability and Work Performance

In Table 2 the results of the linear regression analysis are presented, with work ability and work performance as dependent variables. The total variance of work ability explained by the model was 42 %, F(12,104) = 6.34, *p* = 0.001. Younger age, better perceived general health and higher beliefs of pain self-efficacy were associated with higher work ability in workers who stayed at work with CMP. Work ability was not associated with pain intensity, fear avoidance beliefs, physical work demand category, full-time work, control over work tasks and work satisfaction. The total variance of work performance explained by the model was 37 %, F(12,103) = 4.97, *p* = 0.001. Younger age, higher beliefs of pain self-efficacy, lower physical work demand category and having a part-time job were associated with a higher work performance. Work performance was not associated with pain intensity, general health perception, fear avoidance beliefs, control over work tasks and work satisfaction.

**Table 2:** Hierarchical multiple regression analysis with work ability and work performance as dependent variables

Model	Work ability				Work performance			
	R^2^	Change in R^2^	Standardized ß	*p* value	R^2^	Change in R^2^	Standardized ß	*p* value
1	0.029				0.034			
Age			−0.140	0.136			−0.144	0.125
Gender			−0.083	0.374			−0.099	0.293
2	0.110	0.080			0.063	0.028		
Age			−0.156	0.085			0.153	0.102
Gender			−0.101	0.263			−0.111	0.234
Pain intensity			−0.285	**0.002**			−0.169	0.069
3	0.358	0.248			0.214	0.152		
Age			−0.168	**0.033**			−0.165	0.059
Gender			−0.084	0.299			−0.111	0.218
Pain intensity			−0.045	0.601			−0.005	0.954
General health perception			0.231	**0.012**			0.026	0.795
Fear avoidance beliefs			−0.034	0.687			0.053	0.572
Pain self efficacy beliefs			0.388	**0.000**			0.424	**0.000**
4	0.423	0.065			0.367	0.152		
Age			−0.183	**0.020**			−0.185	**0.026**
Gender			−0.150	0.147			0.112	0.301
Pain intensity			−0.006	0.941			−0.015	0.872
General health perception			0.217	**0.023**			−0.049	0.624
Fear avoidance beliefs			0.013	0.873			0.097	0.277
Pain self efficacy beliefs			0.423	**0.000**			0.458	**0.000**
Light physical work			−0.099	0.267			−0.213	**0.024**
Moderate physical work			−0.052	0.555			0.015	0.866
(Very) heavy physical work			−0.142	0.100			−0.287	**0.002**
Full-time versus part-time work			0.117	0.231			−0.215	**0.038**
Control over work tasks			−0.125	0.158			0.110	0.238
Work satisfaction			−0.099	0.241			−0.164	0.067

A *p* value <0.05 was considered significant and was indicated bold

Logistic regression revealed that high work ability was explained by age (OR = 0.90; 95 % CI: 0.84–0.97; *p* = 0.007), general health perception (OR = 1.04; 95 % CI: 1.00–1.07; *p* = 0.036) and pain self-efficacy (OR = 1.15; 95 % CI:1.05–1.25; *p* = 0.002). High work performance was only associated with pain self-efficacy beliefs (OR 1.11; 95 % CI 1.04–1.19; *p* = 0.003). This means that with every year older, the odds of having high work ability decrease 1.11 times (10 years older decreases the odds 2.84 times). With every unit higher on the RAND-36 general health perception subscale (range 0–100), the odds of having high work ability increase 1.04 times. With every unit higher on the PSEQ (range 0–60), the odds of having high work ability increase 1.15 times (10 points higher increase the odds 4.05 times), and the odds of reporting high work performance increase 1.11 times (10 points higher increase the odds 2.84 times). All other independent variables were not associated with high work ability and work performance.

## Discussion

The aim of this study was to assess self-reported work ability and work performance of workers who stay at work despite CMP, and to explore associated variables. Most workers with CMP report poor to moderate work ability and moderate work performance. Younger age, better perceived general health and higher beliefs of pain self-efficacy were associated with higher work ability. Younger age, higher beliefs of pain self-efficacy, lower physical work demand category and having a part-time job were associated with higher work performance.

The observed rates of work ability in our study were lower compared to another study investigating people with CMP [20]. In that study, the sample was younger (mean age 42, vs 48 years in the present study), which is a plausible explanation for the higher observed work ability. In comparison, healthy workers rated their current work ability on average at 88 % [56] and 79 % [57], which was, after being transformed to a 0–10 scale, 1.7 and 0.8 point higher than the work ability reported in our study. The rates of work performance observed in our study were in accordance with the results of Bernaards et al. [58], who also used the HPQ in workers with neck and upper limb symptoms, and others [4, 12]. Contrary to the latter findings, in our study no association was observed between pain severity and work performance. The reason for this might be that, contrary to others, we included personal variables into the regression analysis, which might have moderated the effect of pain. The mean work performance of a healthy reference group was 8.6 ± 1.2 [59], which is on average 0.9 point higher compared to the workers with CMP. Our results suggest that staying at work with CMP is, on average, associated with reduced performance. Compared to absent workers due to CMP, workers who stay at work with CMP reported much higher work ability and work performance (WAI single item: 7.1 vs 3.8; HPQ: 7.7 vs 4.7) [60].

Reduced work performance is also seen in populations without a chronic health condition [4, 59]. Therefore, reduced work performance is not necessarily attributed to a chronic health condition. When presenteeism is assessed, a comparison with a healthy non-pain reference group is recommended: considering work performance of 100 % as the norm may lead to underestimation of the work performance of workers with CMP or other health problems. Workers with CMP have indicated that when they experience that their work performance or quality of work would decrease beyond acceptable levels, they would decide to call in for sick leave [61, 62]. This concern of being able to meet the job demands may explain the relatively low declined work performance of these workers with CMP: they continue work until they experience that job demands are no longer met.

Although presenteeism is described as an important factor for productivity loss, in some studies it has been observed that workers with a chronic health condition generally perform well while being at work [27]. Continuing work with CMP can even be beneficial and entail a therapeutic effect [62]. Even so, working with pain is considered by some as a non-desirable behavior which even could be harmful [7, 63]. Reduced work performance due to health problems such as CMP is not desirable, but the alternative of work absence may be even worse, reflected by declining return-to-work perspectives as the length of work absence increases [64, 65]. Presenteeism “should not necessarily be interpreted as a negative thing, either for the individual or the company” [22]. Staying at work with pain may be regarded as a healthy coping behavior, which will help to maintain the workers’ longstanding participation in work and quality of life [6].

Beliefs of pain self-efficacy were strongly associated with work ability and work performance. Pain self-efficacy reflects “how much effort people will expend and how long they will persist in the face of obstacles and aversive experiences” [48]. High self-efficacy beliefs may facilitate behavior which improves work ability and work performance. At the workplace, beliefs of pain self-efficacy seem to have a moderating effect on work ability and work performance. Workers with high beliefs of pain self-efficacy seem to be able to maintain work ability and work performance the best. In vocational rehabilitation, beliefs of pain self-efficacy might be an important treatment mediator, by which increased work ability and/or work performance can be achieved. Further research is needed to confirm this.

Older workers with CMP are at risk of having reduced work ability and performance, which has also been observed in other studies [11, 59, 66, 67]. To maintain work ability and performance in the workforce, extra attention to this group is needed. Pain intensity and fear avoidance beliefs were not related to either work ability or work performance; our study provided evidence to not recommend the use of these variables to maintain work productivity of workers with CMP. Contrary to other findings [27], but in accordance with another study [68], in our study full-time work was negatively associated with work performance. Possibly, in part-time employment, workers can better compensate for reduced capability. Evidence on the effect of part-time versus full-time employment is not robust. In accordance with our study, others have also observed reduced work ability [20] and work performance in workers performing heavy work [25, 28]. In cases of heavy workload, work performance may be increased by adjustment of work demands or making job accommodations. Control over work tasks was not significantly associated with work ability or work performance, in accordance with others [27]. However, in other studies, job control had a moderating effect on reduced work ability [28], or was associated with work performance [26, 69]. Evidence concerning the relation of work control and work performance is conflicting and needs further attention.

Because the term presenteeism assumes a priori loss of productivity, for employers it may be less attractive to employ people with CMP. However, the present study suggests that remaining at work with CMP does not cause productivity loss in all cases. This might be explained by the term “extensionism”, which has been introduced to describe the phenomenon of working extended hours beyond those expected by the employer, to compensate for reduced productivity [70]. Reduced work performance can be compensated by working extended hours (negative absenteeism). This was confirmed in our study, where actual worked hours exceeded the expected worked hours.

There are some limitations and considerations to this study. Firstly, participants responded to a call in a newspaper. In this design selection bias is inevitable and diminishes the external validity of the results. Secondly, because of the cross-sectional data collection, no causal inferences could be made. Thirdly, comparison of work ability measured on a 0–10 scale with reference values of the WAI was performed after transformation of average WAI-scores into a percentage [56, 57]. Therefore, the reference values are an indication and should be interpreted with caution. Fourthly, the construction of the single WAI question “what is your current work ability compared to lifetime best” implies that older workers are more likely to have had higher work ability in their life, because they might have had an onset of the condition at an age older than the younger workers. This might have resulted in an underestimation of work ability of older workers compared to younger workers. However, across many studies on work ability (using the complete WAI), older age was related to lower workability too [11, 67]. Although the mean reported work performance in our study was lower compared to reference values of healthy controls, 81 % of the workers rated their work performance as equal or better compared to their co-workers. When work performance in our study was determined on comparison with workers in similar jobs, it would have exceeded reference values of healthy controls. This illustrates that it matters which instrument is used to measure work performance. Estimates of reduced productivity at work vary considerable according to the instrument chosen [71, 72].

### Clinical Implications

In our study we selected a group of workers with CMP who remained at work without sick leave. Therefore, the generizability of the results to workers with CMP on partly sick leave may be limited. Our results suggest that a subgroup of workers with CMP can stay at work without reduced work ability or work performance, especially when they have high beliefs of pain self-efficacy. In our study it was not possible to make causal inferences, so it is unclear whether these workers have high pain self-efficacy beliefs because they work, or whether they work because of high pain self-efficacy beliefs. It is unclear whether we are able to train self-efficacy with return to work as a result, or whether self-efficacy will be improved from the moment people are placed in work. Longitudinal studies are needed to answer this question. Because work performance in workers with CMP is reduced, intervention programs on CMP at work should focus not only on absenteeism, but on presenteeism as well. Staying at work while suffering from CMP is favorable for sustainable work participation, but is not always obvious to achieve. Our results further show that not the pain itself, but personal and work-related factors relate to work ability and work performance. Work ability may be improved by promoting general health perception and pain self-efficacy beliefs. Work performance may be improved by promoting pain self-efficacy beliefs and part-time employment, and by reducing physical work demands.

## References

[1] Dagenais S, Caro J, Haldeman S (2008). A systematic review of low back pain cost of illness studies in the United States and internationally. Spine J.

[2] Lambeek LC, van Tulder MW, Swinkels IC, Koppes LL, Anema JR, van Mechelen W. The trend in total cost of back pain in the Netherlands in the period 2002–2007. Spine (Phila Pa 1976). 2011;36(13):1050–8. 10.1097/BRS.0b013e3181e70488 21150697

[3] Alavinia ASM (2008). The effect of work on health and work ability [dissertation].

[4] Langley P, Muller-Schwefe G, Nicolaou A, Liedgens H, Pergolizzi J, Varrassi G (2010). The impact of pain on labor force participation, absenteeism and presenteeism in the European union. J Med Econ.

[5] Picavet HS, Schouten JS (2003). Musculoskeletal pain in the Netherlands: prevalences, consequences and risk groups, the DMC(3)-study. Pain.

[6] Waddell G, Burton AK (2006). Is work good for your health and well-being?.

[7] McKevitt C, Morgan M, Dundas R, Holland WW (1997). Sickness absence and ‘working through’ illness: a comparison of two professional groups. J Public Health Med.

[8] Turk DC, Okifuji A (2002). Psychological factors in chronic pain: evolution and revolution. J Consult Clin Psychol.

[9] Tuomi K, Ilmarinen J, Jahkola A, Katajarinne L, Tulkki A (1998). Work ability index, 2nd revised ed.

[10] Alavinia SM, de Boer AG, van Duivenbooden JC, Frings-Dresen MH, Burdorf A (2009). Determinants of work ability and its predictive value for disability. Occup Med (Lond).

[11] Ilmarinen J (2009). Work ability—a comprehensive concept for occupational health research and prevention. Scand J Work Environ Health.

[12] McDonald M, DiBonaventura M, Ullman S (2011). Musculoskeletal pain in the workforce: the effects of back, arthritis, and fibromyalgia pain on quality of life and work productivity. J Occup Environ Med.

[13] Burton WN, Chen CY, Conti DJ, Schultz AB, Pransky G, Edington DW (2005). The association of health risks with on-the-job productivity. J Occup Environ Med.

[14] Stewart WF, Ricci JA, Chee E, Morganstein D (2003). Lost productive work time costs from health conditions in the United States: results from the American productivity audit. J Occup Environ Med.

[15] Hemp P. Presenteeism: at work—but out of it. Harv Bus Rev. 2004;82(10):49–58. 15559575

[16] Collins JJ, Baase CM, Sharda CE, Ozminkowski RJ, Nicholson S, Billotti GM (2005). The assessment of chronic health conditions on work performance, absence, and total economic impact for employers. J Occup Environ Med.

[17] van Leeuwen MT, Blyth FM, March LM, Nicholas MK, Cousins MJ (2006). Chronic pain and reduced work effectiveness: the hidden cost to Australian employers. Eur J Pain.

[18] Loeppke R, Taitel M, Richling D, Parry T, Kessler RC, Hymel P (2007). Health and productivity as a business strategy. J Occup Environ Med.

[19] Hagberg M, Tornqvist EW, Toomingas A (2002). Self-reported reduced productivity due to musculoskeletal symptoms: associations with workplace and individual factors among white-collar computer users. J Occup Rehabil.

[20] Holtermann A, Hansen JV, Burr H, Sogaard K (2010). Prognostic factors for long-term sickness absence among employees with neck-shoulder and low-back pain. Scand J Work Environ Health.

[21] Brouwer WB, Meerding WJ, Lamers LM, Severens JL (2005). The relationship between productivity and health-related QOL: an exploration. Pharmacoeconomics.

[22] Mannion AF, Horisberger B, Eisenring C, Tamcan O, Elfering A, Muller U (2009). The association between beliefs about low back pain and work presenteeism. J Occup Environ Med.

[23] Larsson A, Karlqvist L, Gard G (2008). Effects of work ability and health promoting interventions for women with musculoskeletal symptoms: a 9-month prospective study. BMC Musculoskelet Disord.

[24] Marks R, Allegrante JP, Lorig K (2005). A review and synthesis of research evidence for self-efficacy-enhancing interventions for reducing chronic disability: implications for health education practice (part II). Health Promot Pract.

[25] Meerding WJ, IJzelenberg W, Koopmanschap MA, Severens JL, Burdorf A (2005). Health problems lead to considerable productivity loss at work among workers with high physical load jobs. J Clin Epidemiol.

[26] Wynne-Jones G, Buck R, Varnava A, Phillips C, Main CJ (2009). Impacts on work absence and performance: what really matters?. Occup Med (Lond).

[27] van den Heuvel SG, Geuskens GA, Hooftman WE, Koppes LL, van den Bossche SN (2010). Productivity loss at work; health-related and work-related factors. J Occup Rehabil.

[28] van den Berg TI, Robroek SJ, Plat JF, Koopmanschap MA, Burdorf A (2011). The importance of job control for workers with decreased work ability to remain productive at work. Int Arch Occup Environ Health.

[29] Central Statistics Office. Sickness absence stabilising last years. In Dutch: Centraal bureau voor statistiek. ziekteverzuim laatste jaren stabiel. CBS-publications; 2010.

[30] Livanos I. Sickness absence a Pan-European study. 2010. http://mpra.ub.uni-muenchen.de/22627. Accessed 6 Oct 2011.

[31] Peduzzi P, Concato J, Kemper E, Holford TR, Feinstein AR (1996). A simulation study of the number of events per variable in logistic regression analysis. J Clin Epidemiol.

[32] Ahlstrom L, Grimby-Ekman A, Hagberg M, Dellve L (2010). The work ability index and single-item question: associations with sick leave, symptoms, and health—a prospective study of women on long-term sick leave. Scand J Work Environ Health.

[33] Gould R, Ilmarinen J, Jarvisalo J (2008). Dimensions of work ability.

[34] Neupane S, Miranda H, Virtanen P, Siukola A, Nygard CH (2011). Multi-site pain and work ability among an industrial population. Occup Med (Lond).

[35] Kessler RC, Barber C, Beck A, Berglund P, Cleary PD, McKenas D (2003). The world health organization health and work performance questionnaire (HPQ). J Occup Environ Med.

[36] Kessler RC, Ames M, Hymel PA, Loeppke R, McKenas DK, Richling DE (2004). Using the world health organization health and work performance questionnaire (HPQ) to evaluate the indirect workplace costs of illness. J Occup Environ Med.

[37] Engers AJ, Koke A, Torenbeek M (2007). Nederlandse dataset pijnrevalidatie.

[38] Von Korff M, Ormel J, Keefe FJ, Dworkin SF (1992). Grading the severity of chronic pain. Pain.

[39] Jensen MP, Karoly P, Braver S (1986). The measurement of clinical pain intensity—a comparison of 6 methods. Pain.

[40] Childs JD, Piva SR, Fritz JM. Responsiveness of the numeric pain rating scale in patients with low back pain. Spine (Phila Pa 1976). 2005;30(11):1331–4. 10.1097/01.brs.0000164099.92112.29 15928561

[41] Pollard CA (1984). Preliminary validity study of pain disability index. Percept Mot Skills.

[42] Tait RC, Chibnall JT, Krause S (1990). The pain disability index: psychometric properties. Pain.

[43] van der Zee KI, Sanderman R. Measuring general health with the RAND-36: A manual. In Dutch: Het meten van de algemene gezondheidstoestand met de RAND-36: Een handleiding. Groningen: Noordelijk Centrum voor Gezondheidsvraagstukken, NCG; 1993.

[44] Goubert L, Crombez G, Van Damme S, Vlaeyen JW, Bijttebier P, Roelofs J (2004). Confirmatory factor analysis of the Tampa scale for kinesiophobia: invariant two-factor model across low back pain patients and fibromyalgia patients. Clin J Pain.

[45] Lousberg R, Van Breukelen GJ, Groenman NH, Schmidt AJ, Arntz A, Winter FA (1999). Psychometric properties of the multidimensional pain inventory, Dutch language version (MPI-DLV). Behav Res Ther.

[46] Vlaeyen JW, Kole-Snijders AM, Boeren RG, van Eek H (1995). Fear of movement/(re)injury in chronic low back pain and its relation to behavioral performance. Pain.

[47] Nicholas MK. In: Self-efficacy and chronic pain. Paper presented at the annual conference of the British psychological society. St Andrews; 1989.

[48] Nicholas MK (2007). The pain self-efficacy questionnaire: taking pain into account. Eur J Pain.

[49] de Zwart BC, Frings-Dresen MH, van Duivenbooden JC (2002). Test-retest reliability of the work ability index questionnaire. Occup Med (Lond).

[50] van Veldhoven M. Psychosocial job demand and work stress [dissertation]. In Dutch: Psychosociale arbeidsbelasting en werkstress. University of Groningen, The Netherlands; 1996.

[51] U.S. Department of Labor. The revised handbook for analyzing jobs. 4th ed. Indianapolis: JIST Works Inc; 1991.

[52] Bos J, Kuijer PP, Frings-Dresen MH (2002). Definition and assessment of specific occupational demands concerning lifting, pushing, and pulling based on a systematic literature search. Occup Environ Med.

[53] Soer R, van der Schans CP, Geertzen JH, Groothoff JW, Brouwer S, Dijkstra PU (2009). Normative values for a functional capacity evaluation. Arch Phys Med Rehabil.

[54] Statistical package for the social sciences (SPSS statistics 18.0.3). Chicago, IL: SPSS Inc.; September 2010.

[55] Brouwer S, Krol B, Reneman MF, Bultmann U, Franche RL, van der Klink JJ (2009). Behavioral determinants as predictors of return to work after long-term sickness absence: an application of the theory of planned behavior. J Occup Rehabil.

[56] Blangsted AK, Sogaard K, Hansen EA, Hannerz H, Sjogaard G (2008). One-year randomized controlled trial with different physical-activity programs to reduce musculoskeletal symptoms in the neck and shoulders among office workers. Scand J Work Environ Health.

[57] Lin S, Wang Z, Wang M (2006). Work ability of workers in western china: reference data. Occup Med (Oxford).

[58] Bernaards CM, Proper KI, Hildebrandt VH (2007). Physical activity, cardiorespiratory fitness, and body mass index in relationship to work productivity and sickness absence in computer workers with preexisting neck and upper limb symptoms. J Occup Environ Med.

[59] Deckersbach T, Stange JP, Nierenberg AA. Norms for performance in the workplace in healthy people: data from the national comorbidity survey replication study. CNS Spectr. 2011 Jul 1 [Epub ahead of print]. 10.1017/S1092852912000314 24725499

[60] de Vries HJ, Reneman MF, Groothoff JW, Geertzen JH, Brouwer S. Workers who stay at work despite chronic nonspecific musculoskeletal pain: do they differ from workers with sick leave? J Occup Rehabil. 2012 Mar 28 [Epub ahead of print]. 10.1007/s10926-012-9360-6 PMC3484275 22454300

[61] Shaw WS, Huang YH (2005). Concerns and expectations about returning to work with low back pain: identifying themes from focus groups and semi-structured interviews. Disabil Rehabil.

[62] de Vries HJ, Brouwer S, Groothoff JW, Geertzen JH, Reneman MF (2011). Staying at work with chronic nonspecific musculoskeletal pain: a qualitative study of workers’ experiences. BMC Musculoskelet Disord.

[63] Rosvold EO, Bjertness E (2001). Physicians who do not take sick leave: hazardous heroes?. Scand J Public Health.

[64] Krause N, Frank JW, Dasinger LK, Sullivan TJ, Sinclair SJ (2001). Determinants of duration of disability and return-to-work after work-related injury and illness: challenges for future research. Am J Ind Med.

[65] Viikari-Juntura E, Kausto J, Shiri R, Kaila-Kangas L, Takala EP, Karppinen J, et al. Return to work after early part-time sick leave due to musculoskeletal disorders: a randomized controlled trial. Scand J Work Environ Health. 2011 Oct 27 [Epub ahead of print]. 10.5271/sjweh.3258 22033811

[66] van den Berg TI, Elders LA, de Zwart BC, Burdorf A (2009). The effects of work-related and individual factors on the work ability index: a systematic review. Occup Environ Med.

[67] Koolhaas W, van der Klink JJ, Groothoff JW, Brouwer S. Towards a sustainable healthy working life: associations between chronological age, functional age and work outcomes. Eur J Public Health. 2011 Mar 31 [Epub ahead of print]. 10.1093/eurpub/ckr035 21459842

[68] Wynne-Jones G, Main CJ (2011). Overcoming pain as a barrier to work. Curr Opin Support Palliat Care.

[69] Alavinia SM, van den Berg TI, van Duivenbooden C, Elders LA, Burdorf A (2009). Impact of work-related factors, lifestyle, and work ability on sickness absence among Dutch construction workers. Scand J Work Environ Health.

[70] Hilton MF, Sheridan J, Cleary CM, Whiteford HA (2009). Employee absenteeism measures reflecting current work practices may be instrumental in a re-evaluation of the relationship between psychological distress/mental health and absenteeism. Int J Methods Psychiatr Res.

[71] Mattke S, Balakrishnan A, Bergamo G, Newberry SJ (2007). A review of methods to measure health-related productivity loss. Am J Manag Care.

[72] Zhang W, Bansback N, Anis AH (2011). Measuring and valuing productivity loss due to poor health: a critical review. Soc Sci Med.

